# Mechanical Appliances in Operative Dentistry

**Published:** 1875-01

**Authors:** M. N. Chappell


### MECHANICAL APPLIANCES IN OPERATIVE DENTISTRY.

BY DR. M. N. CHAPPELL.

*Read before the Indiana State Dental Society, June,* 1874.

*Gentlemen:* The duty assigned me to introduce the subject of "Mechanical Appliances in Operative Dentistry," gives me pleasure to present favorable pleadings for the same.

Appliances have their associates or assistants. I therefore to some extent place them in classes, beginning with the rubber dam, assisted by the clamps, waxed silk, guilling twine, holders, weights, punches and wedges.

I use the dam in every case of filling, preparation of the cavities, or in treating pulps. The medium rubber is preferable for all purposes. Before applying the dam, I examine and and cleanse the crowns which I wish to cover, unless there be deposits of calculi that will assist in retaining the dam. If the teeth are crowded, pass a waxed twine between them if possible, or if too close to admit the thread or twine, I apply the separators or wedge, until there is room sufficient to admit the twine and rubber. There are cases that the passing of the rubber between the teeth is unnecessary, which I will explain in time. For the front teeth small holes cut with the punch on the end of box wood block, sixteenth to quarter inch apart. Stretch the rubber over the tooth, or teeth, with the edges turned under the margin of the gum, long wedges of orange or other hard wood facilitates the forcing of the rubber between the teeth, also the twine firmly grasped carries the dam to its place, if necessary, with a well waxed twine tie the same down the neck of the tooth, as far as it will go, leave the strings long to fasten to the holder, if in the upper teeth, or if in the lower teeth, to fasten the weights to, if desired. This management of the surplus twine prevents the apron holder from changing the dam to let in moisture, be careful that the holder does not draw to tight. Seldom will the clamps be needed on cuspids or incisors, occasionally we use them on bicuspids.

For molars cut a larger hole than for other teeth. Place the jaws of the clamp through the rubber, adjust the clamp forceps, folding the apron over the forceps so you can see the jaws of the clamp, place the same on the tooth, remove the forcep and with a burnisher raise the rubber over the jaws. It adjusts itself to the neck on buccal or lingual surface, if you wish to operate in crown cavities only, this will be sufficient; without passing down between the teeth, as the dam is complete, if necessary or convenient, force the rubber between them as in front teeth, and tie the same.

I adjust the dam on molars in nearly every case in this way. If in operating in approximal cavities, place the clamp as before on the posterior tooth I wish exposed, if the anterior tooth should be crowded, then with a small wire clamp adjusted in the forceps, place the points in the hole for the tooth, bring forward and place the clamp between the teeth. It holds the rubber so you can with wedge and twine complete the dam. You are now ready to shape the cavity. Make critical examinations and thorough operations which cannot be done without the tooth is absolutely dry. The clamps on the molars cannot be dispensed with since their use and application is so well understood, and the dam a universal success.

The burring engine, with its various attachments—burs, drills, corundum discs and points, Arkansas, Hindoostan, and Scotch stone, leather, turned wood discs and points, burnishers and buffers, for each and every case a variety is required. The corundum discs have taken the place of the file to a great extent; more rapidly is the work done, and with better results. The small thin wheel points I use in opening and finishing fissures and beveling the edges of cavities. In case the fissure is deep the bur will have to be used, the small round one is in greatest demand completing the work in the fissures, cutting canals, or under cuts to secure the filling, also rounding out all acute angles, beveling the edge of retaining pits which are cut with the diamond point drill. After the filling in crown or compound cavities is condensed, all parts full, the corundum discs, wheels and cones, cut down to a surface more rapidly that the finishing burs or files, complete the finish with fine stones, wooden points, burnisher and buffer with rouge.

Superficial decay I cut away with corundum and finish with fine stones, wood discs or points with pumice. In green stain I use the wood points and pumice, unless it approaches superficial decay, then I treat it as such.

Great care is required in using burs, discs, etc., to not allow the point to "jump" and come in contact with the dam or mouth. In small approximal cavities, or fillings, where you gain access by wedging, the engine cannot be used to that extent as is required where the caries is deep seated and portions of tooth gone.

The napkin is not dispensed with since the use of the rubber dam has become so general, but welcome in nearly every case we have, to lay over the nose or mouth of the patient to prevent the inhaling of their breath, in holding away the lips, in forming a partial dam, in examinations or excavations before applying the rubber dam, also folded to lay under the rubber over the chin or across the mouth, it absorbs the saliva, keeps the chin dry and renders some degree of comfort to the patient, renew often if necessary when the saliva flows freely.

Wedges of hickory, orange, or other hard wood, become necessary, when the teeth are too close for adjusting the rubber dam, removing decay and filling and finising the same is required. Quick wedging I believe to be the best, start two or more one at the point, one at the neck of the tooth and one mid-way, give time for the membrane and tissues to give way, employing the time on teeth that require no wedging; remove all but the lower wedge, when there is space sufficient to work in finishing the filling over the edge of the cavity and leaving the same convex, the natural shape of the tooth; you might break into the cavity, with chisel or file, prepare it so it would retain a filling for the time being without wedging, but as so much depends on the finish of a filling for the subsequent preservation of the tooth; the space is necessary and must be made, and wedging is the best means to be employed. I have used the Jarvis separators for several weeks, They are intended to take the place of the wooden wedge, are to be used in localities where wedging is nearly impossible. In some cases they work well; I am not prepared to praise or condemn them. I have been using a strong wire clamp resembling the shape of a horse shoe, placed between the teeth, the spring force in many cases causes sufficient space between the molars, or other teeth to admit the twine and dam, or the holding wedge at the gum, when I find space sufficient remove the clamp and proceed.

I continue the use of the mallet, and mallet pluggers, of my own design, believing that I do much better work since learning to point and polish my instruments, keeping them in good order, the serrations are ten points to the sixteenth of an inch —lineal. When the serrations are coarser, deep and sharp, the cohesive surface is very imperfect, the gold crystals are cut or broken and the foil "balls up," and disappointment will be the rule. The welding is difficult, the filling weak by interstices, the margins imperfect, and in many cases the walls damaged by the points, this trouble is obviated by fine serrations and where the force is direct, finer serrations are still preferable, the serrations act as steps to retain or support the crystals in receiving the force and this principle must be recognized all the way in making a compact and thorough filling. My favorite patterns are foot shape, with distinct heels at angles from ten to thirty degrees also of double heels at right angles, curved at shank rights and lefts foot shape, and one concave from heel to toe, fluted serrations lengthwise. This instrument I use for condensing the last layers over convex surfaces, a small double curve straight point for filling retaining pits. With these I can fill all the cavities in any tooth, including the posterior molar distal surface.

The mallet, of lead with rubber covering, seven oz. weight, with foil carrier attached to the handle near the mallet. I do my own feeding and malleting in most cases, I think it can be done better and time saved, controlling the instrument with the left hand, and feeding and malleting with the right hand.

The gold screws for securing fillings are used where the strain on the retaining points of the filling will be great, such as building down abraded crowns, and in many cases of compound fillings. I use them as posts set in any surface of the crowns of teeth, in correcting irregularities, either to attach ligatures, rubber bands, etc. When their use as posts is not required any longer, cut them off and polish or take out and fill with foil. Many cases of setting crowns on roots instead of using the old style wooden pivot teeth, use plain plate teeth, root capped and long screws extending through, and allowing the plate to be attached by the screws continue welding the gold capping over the margins of plate, uniting with the screws. Finish up the palatine surface, shape of natural tooth, welding then the labial joint completing a substantial as well as beautiful operation. In some cases the clamps or twine will not retain the rubber dam on a tooth, owing to the shape of the crown or locality of cavity, by setting a screw in the locality desired, the clamp remains firm, lodging beneath the screw securely. When the filling is complete, cut off the screw and polish down.

The Lawrence file carier fills a very important place in holding short pieces of files at any angle desired in dressing approximal cavities or fillings in molars, holding small wedges for adjusting the dam, also pieces of corundum or wood in dressing teeth in the back part of the mouth or a lingual surface of lower front teeth. Excavators in the shape of spoons, long shank, double edge, hoes and hatchets with oval points are the best. Having them oval at point, in excavating the liability of injuring the pulp is less than if the cutting edges are square also we avoid sharp angles which in many cases the wedge force of an acute angle checks the dentine or enamel by the same principle as cutting glass with the diamond.

Spongoid, a preparation of lint, is the best material I have ever used in drying cavities, dressing pulps, etc., it is pure, neat, and convenient.

There are many details that I might indulge in, but believing this to be an advanced era in operative dentistry, and that each of you are endeavoring to know for himself the benefits derived from having a supply of the improved instruments and appliances and possess them.

You will pardon me for occupying so much of your valuable time, hoping what I have said will cause every member to give his experience with the different appliances.

I believe the services of the dental surgeon are now more fully appreciated by the people, and the success in saving teeth is greatly due to the improvements of which I have spoken, and the professional reputation of the operator is due to his application, and skillful training in the use of "Mechanical Appliances in Operative Dentistry."

